# Baltimore Almshouse

**Published:** 1832-03

**Authors:** 


### HOSPITAL REPORTS.

### BALTIMORE ALMSHOUSE.

*Glandular Tissue, Induresence, Suppuration, and Excrescence of the Testicle, mistaken for Carcinoma.* By T. H. Wright, M.D.*

A young man, twenty-five years of age, wagoner by occupation, entered the Baltimore Almshouse, with disease of the testicle, ensuing, by his report, to severe and neglected gonorrhœa. State of parts on admission: left testis enlarged to thrice its natural size, hard in places, inelastic, not tender to moderate pressure; surface of the gland rough and fibrous, as if traversed by numerous chords or thin bands; scrotum dark brown colour, contracted close around testis, at many points indented by union with the membranes and gland. From the whole front of the testis protruded a mass, of the size of a pullet's egg, of coarse, granular substance, secreting profusely a mixture of sanies and pus. The base of the excrescence was broad and hard, its body elevated, flattened on the top; whole bulk exceeding the size of the testicle; scrotum around the base of the vegetation thick, adherent, and callous feeling. By pressure on the parts, a soft pulpy matter could be made to ooze out at some points where union of the scrotum to the base of the excrescence was incomplete. The funis of the left testicle was thick as a finger, knotted and hard; superficial lymphatics of both groins enlarged; cellular tissue, same seat condensed; inguinal glands swelled and tender to touch. Right testis retracted high, very little altered from natural state, except by adhesion of scrotum, vaginalis, &c. at a few points. Duration of the local disease now, by account of the patient, eleven weeks: parts had never been very painful; occasional sense of stinging and burning in the part, the chief unpleasant feeling.

The man represented the affection of his testicle to have commenced, (while he had gonorrhœa,) by general enlargement of the gland, followed by a dark red swelling on the front part, which slowly gathered, burst, discharged a reddish matter for some time, then large granulations shot out from the cavity of the abscess, and continued to grow and overspread the gland, as they appeared at the time of his admission into the house. The parts had been constantly irritated by riding on horseback in his employment as teamster. The personal appearance and condition of the subject of this affection was strongly marked by signs of constitutional disorder. Form, naturally stout, was considerably emaciated; skin flaccid and sallow; face contracted, look desponding; tongue furred; appetite indifferent; bowels laxative; distressed by flatulence; pulse small and quick; surface dry; sleep irregular and uncomfortable. By all the signs, local and constitutional, the disease of the testicle

* American Journal of Med. S ciences.

seemed to be represented as one of the malignant class of tumors, fungus hematodes, or medullare. Operation in prospect was adverted to, as soon as by rest, regulated diet, and suitable alterative medicines, due preparation of the system was accomplished. The patient was put on the use of Plummer's pill reduced in strength, first with opium added for loose bowels, and free use of the diet drink; regimen, boiled milk, rice, and bread. The part was treated by simple fomentation and poultice.

The general circumstances of this case were so much mended after some time in hospital, (end of second week,) that the patient both looked and felt sensibly better than when admitted; his complexion, spirits, appetite, and strength were greatly improved. The local disease was not materially altered, otherwise than by greater cleanness, a better secretion, more puriform, from the ulcerous surface; better colour of the fungous growth, and less soreness of the parts, particularly of the inguinal indurations. These changes, slight as they were, taken in connexion with the obvious melioration in the general functions, under mere rest and simple treatment, seemed so strongly to contradict the presumption of malignancy in the disease of the part, that as soon as they were displayed in an unequivocal manner, I did not hesitate to recall the opinion first expressed, and to deprecate the contemplated resort to operation, as unwarranted by the present aspect and circumstances of the case. The local disease was now exhibited as essentially the result of irritable inflammation, aggravated constantly by manner of life, exposure, and neglect. It was noticed as probable, also, that the strumous diathesis was present, and concurred to complicate the affection, both by additional irritability in the habit, and the peculiar tendency it is known to impart, to conversions of textures not belonging to the common forms of inflammation. Hence the chronic induration of the tissues, partial, (tuberculoid,) suppurative degenerescence, and subsequent vegetative developement in the part; hence too the manner and form of sympathetic irritation and change in the neighbouring lymphatic and cellular structures.

On this pathology of the affection it was conceived highly probable that perseverance in the same general regimen, with the steady employment of caustic to the part, would accomplish enough to exclude occasion to resort to direct surgery. By maintaining a good state of the general system, and constantly repressing the products of morbid action in the part, the irritability on which that action was sustained would be extinguished, and a healthy process be set up for repair of the organization. Time realized this expectation. The fungus was touched over daily with the solid nitras argenti, and afterwards wrapped in poultice. The application gave little pain, and did not excite inflammation. After a few days' use of the caustic, the bulk of the morbid growth was sensibly lessened; continued employment of it brought the tumor down to near the level of the skin, while the discharge from its surface became good, and the margin of scrotum around the base of excrescence softened, contracted, and was cicatrizing. In a month the mass of vegetation from the testis was demolished, and the general health of the patient reestablished. As the excrescence wasted, the enlargement of the body of the testicle was found to reduce in a gradual manner, and to resume something of its natural form; its regular hardness and fibrous feel also diminished. The treatment by caustic was continued as long as any tendency to excrescence was apparent; the poultice was laid aside when the marks of inflammation were dissipated, subtituted by dressings with ungt. oxyd. hyd. rub. The part finally cicatrized, leaving the body of gland enlarged in some degree, but without evident signs of disease. The secondary affection of the lymphatics, ganglions, and cellular tissue of the groins, subsided so as to be inconsiderable at the time the man left the hospital.

---

### *Muscular Tissue; Calcareous Deposite.**

Among the conversions to which the muscular tissue is liable, suppuration, induresćence, pulpy or lardaceous degeneration, &c., it has been doubted by pathologists of high authority, whether the muscular texture proper was ever the seat of calcareous conversion, or submitted to that change commonly discriminated by the term ossification. The following case may be added to the scanty record on the affirmative side of this question, and appears to furnish, substantially, the kind of proof which the controversy calls for. Yet this case does not fill the vacancy of evidence on that part of the question which demands an instance of ossification in the muscular fibre, wholly primitive in the seat where it is found; originating in, and restricted to the muscular texture, and not produced or propagated to that organism, by extension or enroachment of the ossific process set up in other tissues, to which such a change is easy or common.

A man, about thirty years of age, was brought to the Baltimore Almshouse, January 1831, in a state of low exhaustion, ensuing to the joint influence of long intemperance and much exposure to the rigor of the season. The endeavours to sustain him proved ineffectual, and he died on the third day from his admission. In the examination of this patient when first brought into hospital, it was noticed that one of his legs was very much deformed by morbid enlargement of irregular figure and singular hardness. There was extensive cicatrization on the leg, as if from former sores; but at this time the surface was no where ulcerated. After death, the deformed leg became the subject of examination, and the hard enlargement, inequality, &c. of the limb, was then found to be caused by exostosis of the fibula in its whole extent. Two parallel spines, or ridges of super-ossification, were produced, from the edges of the fibula down its entire length; they were more than an inch deep, and spread outwards from their line of origin, so as to give the

* Ibid. from Dr. Wright's Reports.

fibula the appearance of a long bony trough, wider at top than bottom. Between the anterior spine or ridge, and the tibia, and raised considerably above them both, appeared a bundle or tract of matter, distinct from both bones of the leg, but nearly as solid and bone-like as either in the greater part of its extent. In places this interposed substance was made up of hard and soft matter intermingled, and at those points retained a good deal the colour, texture, &c. of muscular fibre, blended with much earthy matter. In other places, some inches in length, the structure of the part resembled an entire rough bony body. Where the substance under consideration was least solidized, it was very much increased in bulk, forming at two or three points in the length of the leg, large knobs in a semi-converted state, thus showing the cause of the general increase of volume, as well as irregularity of form, noticed in the limb on first inspection of the case. This intermediate osseo-muscular stricture was composed of all the muscles on the anterior aspect of the leg between the bones; the tibialis anticus, extensor proprius, pol. ped. extens., long. digit. ped. &c., all degenerated more or less completely into osseous matter, and fused into a complex mass.

Although it appears probable that super-ossification in the present instance was first set up by the periosteum of the fibula, and was propagated to the inter-osseous muscular tissue, yet the conversion of the muscles does not appear to have been accomplished by direct extension, or mere augmentation of earthy matter from the primary source of deposit. The semi-ossified mass of muscles was distinct and separable in its whole course from the bones of the leg, and, by osseous developement of the fibula inwards, had been pressed up so as to lie above them both. The stimulus or irritation to morbid secretion may have been imparted by similar action in the neighbouring tissues, but the earthy deposit amongst the muscular fibre seems to have been properly the work of its own vessels of nutrition.

